# Supplementary figures and images for: Caveolae and Bin1 form ring-shaped platforms for T-tubule initiation
Source: eLife. 2023 Apr 21;12:e84139. doi: 10.7554/eLife.84139 (PMC10281672; doi:10.7554/eLife.84139)

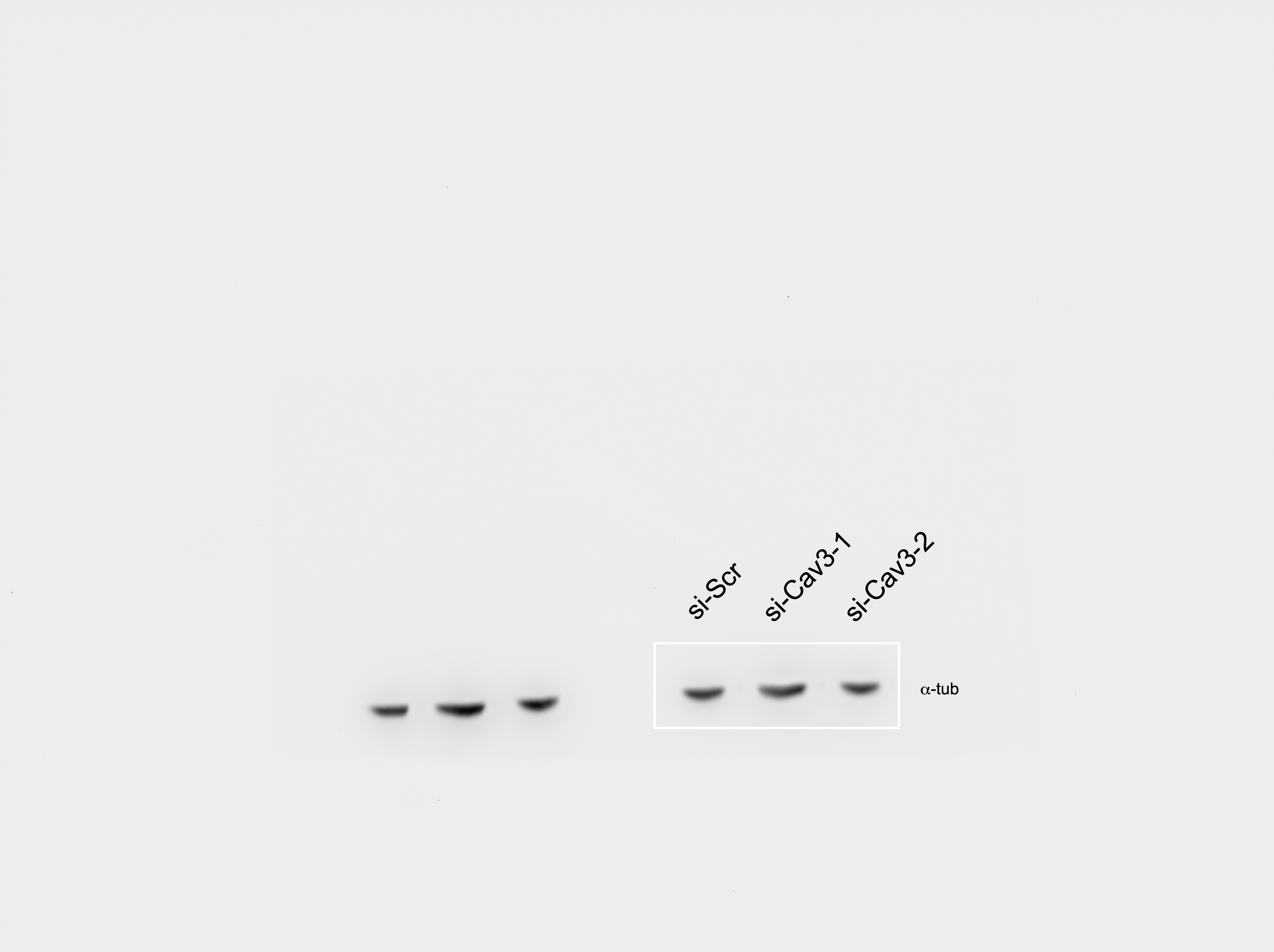

Supplement: Figure 7—source data 3. [file elife-84139-fig7-data3.zip › Figure 7 - source data 3. Western-blot uncropped membranes/alpha tubulin.tif]

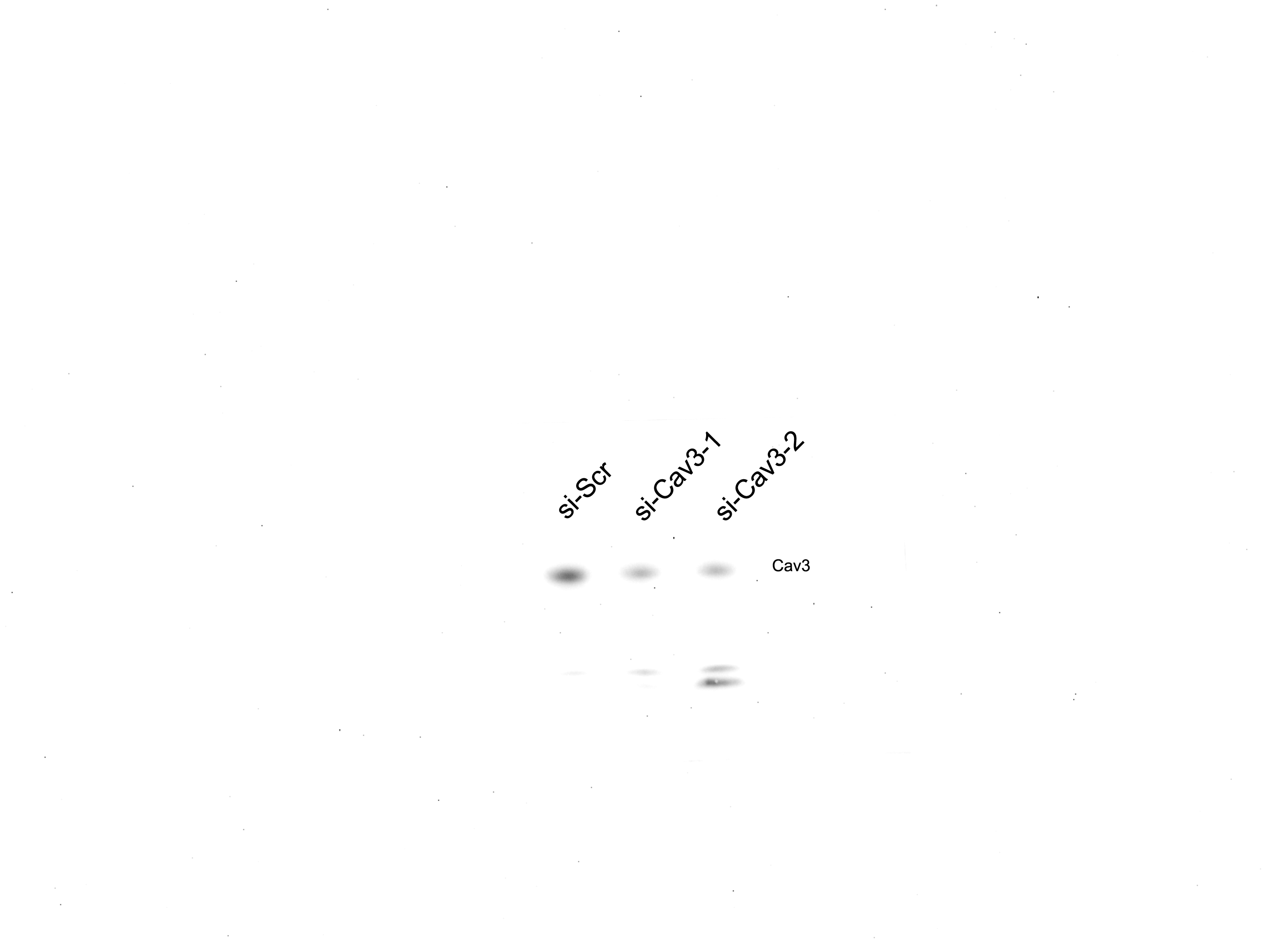

Supplement: Figure 7—source data 3. [file elife-84139-fig7-data3.zip › Figure 7 - source data 3. Western-blot uncropped membranes/Cav1.tif]

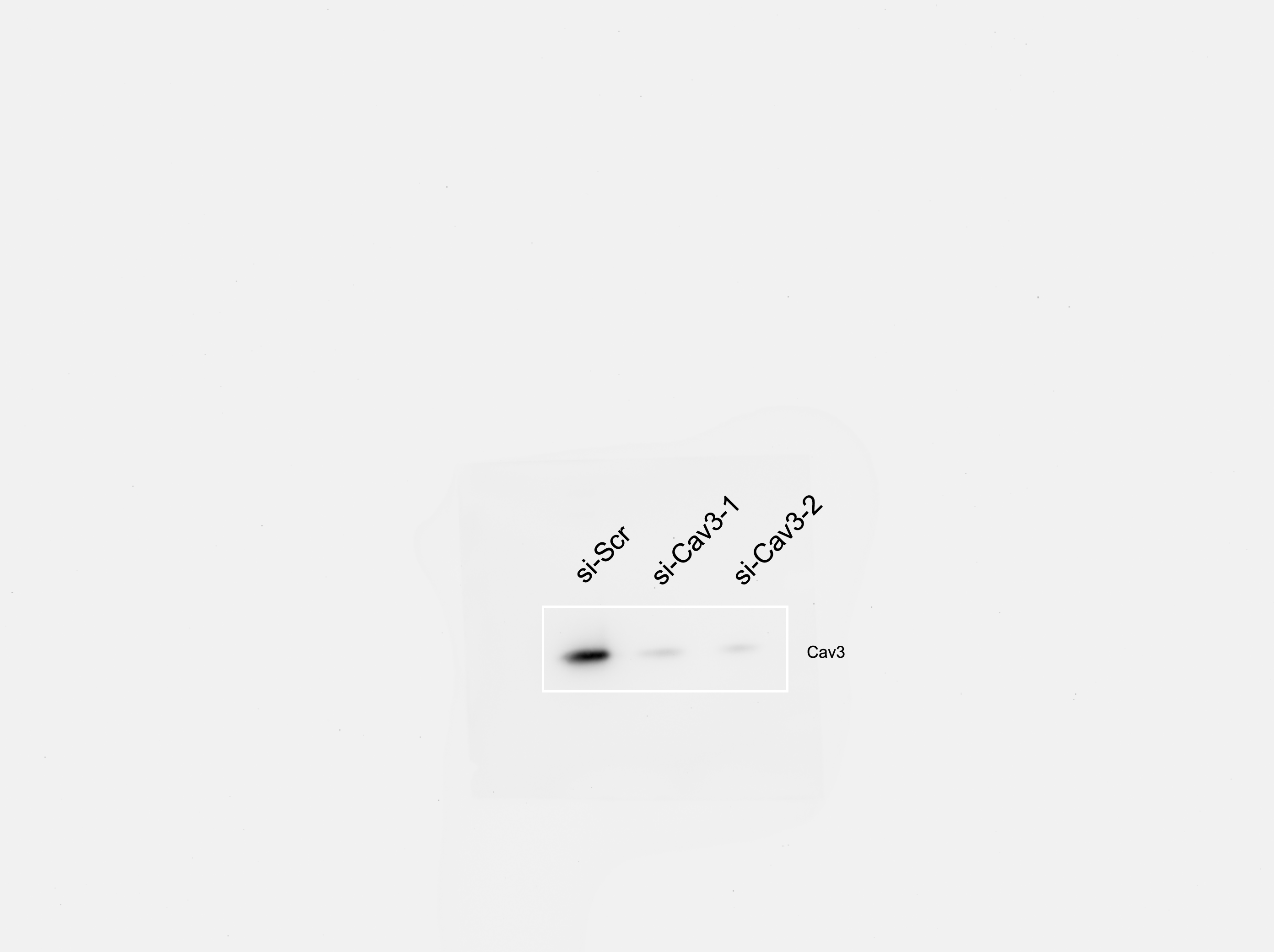

Supplement: Figure 7—source data 3. [file elife-84139-fig7-data3.zip › Figure 7 - source data 3. Western-blot uncropped membranes/Cav3.tif]

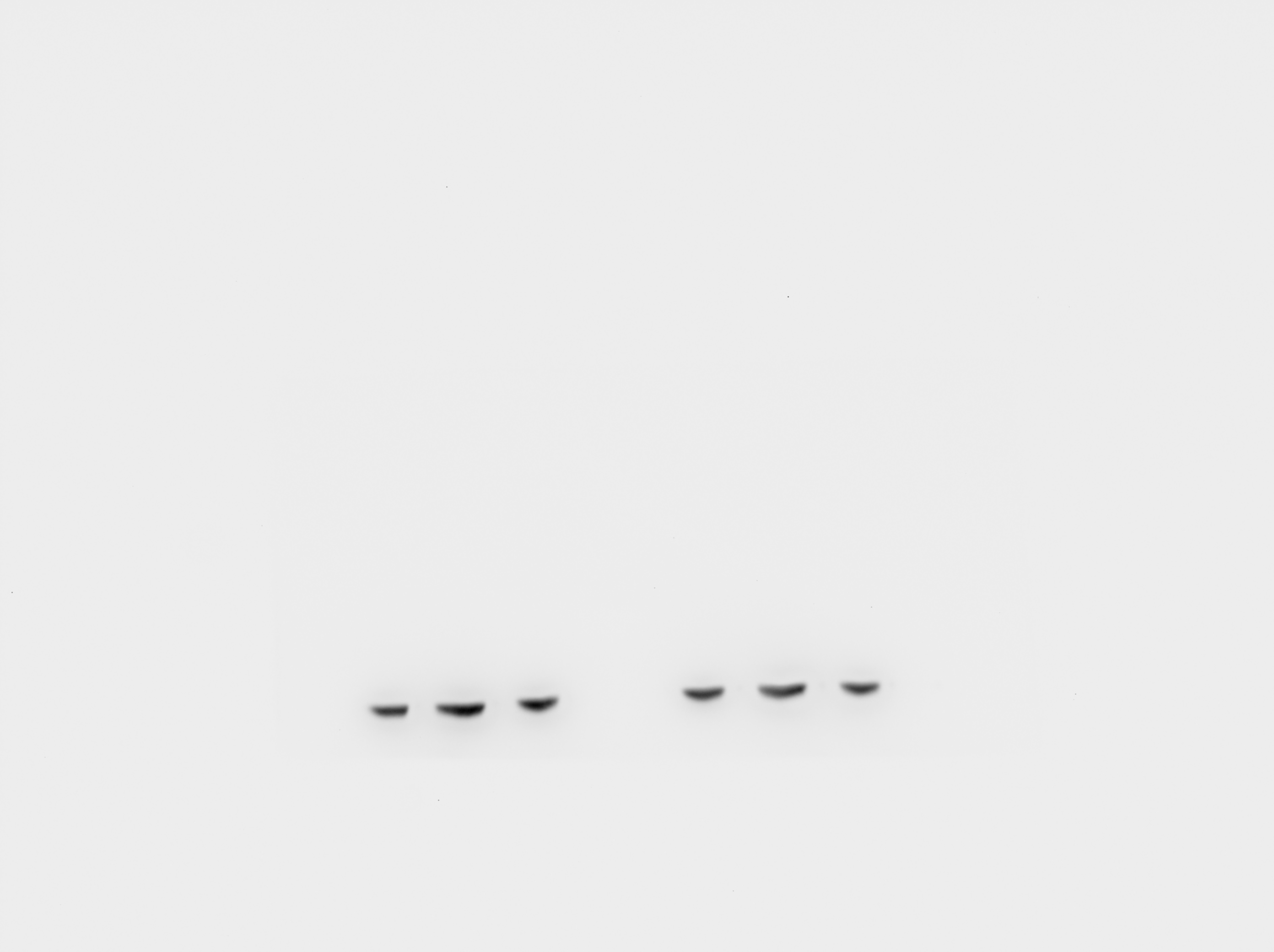

Supplement: Figure 7—source data 3. [file elife-84139-fig7-data3.zip › Figure 7 - source data 3. Western-blot uncropped membranes/Full blot alpha tubulin.tif]

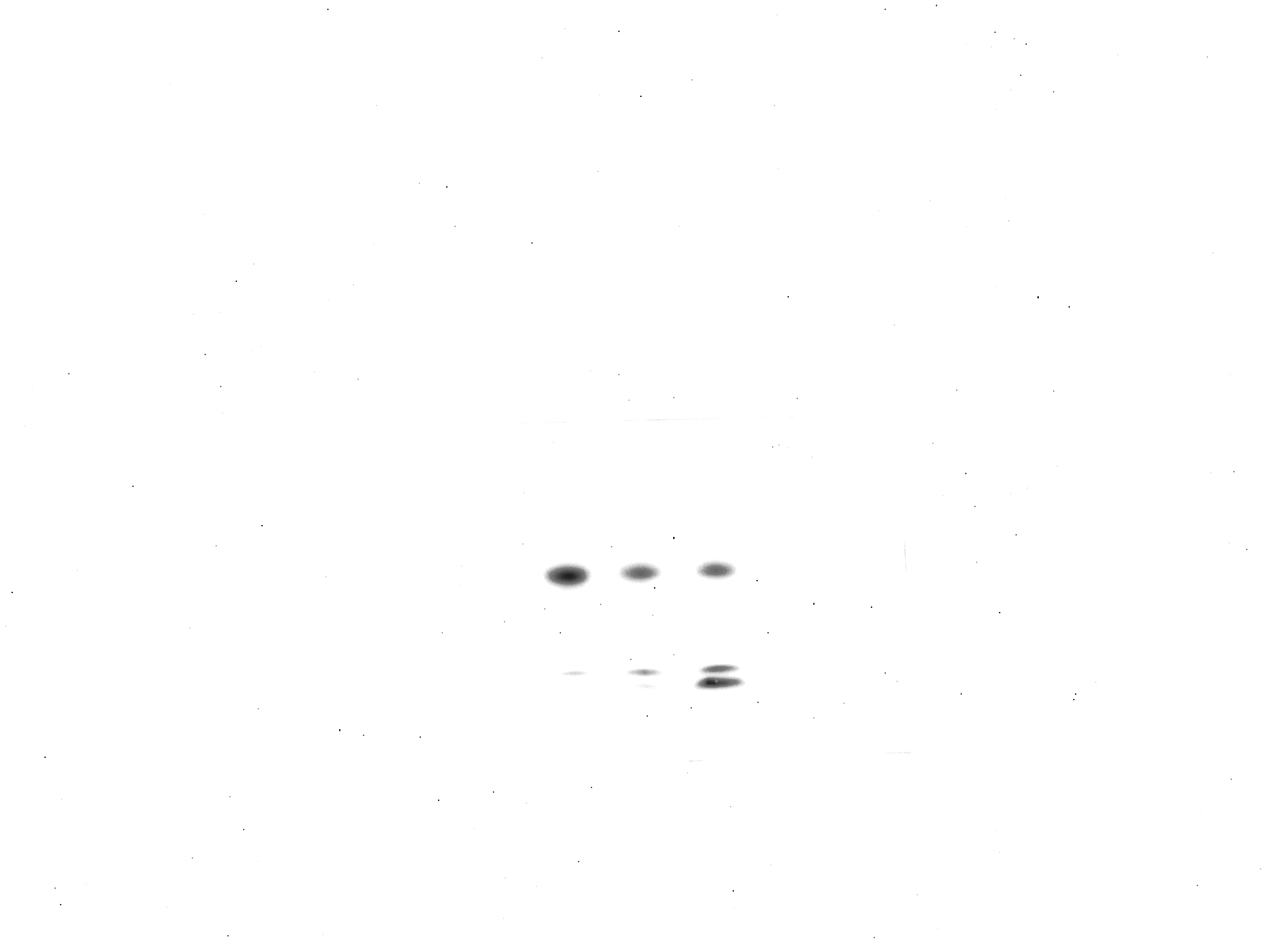

Supplement: Figure 7—source data 3. [file elife-84139-fig7-data3.zip › Figure 7 - source data 3. Western-blot uncropped membranes/Full blot Cav1.tif]

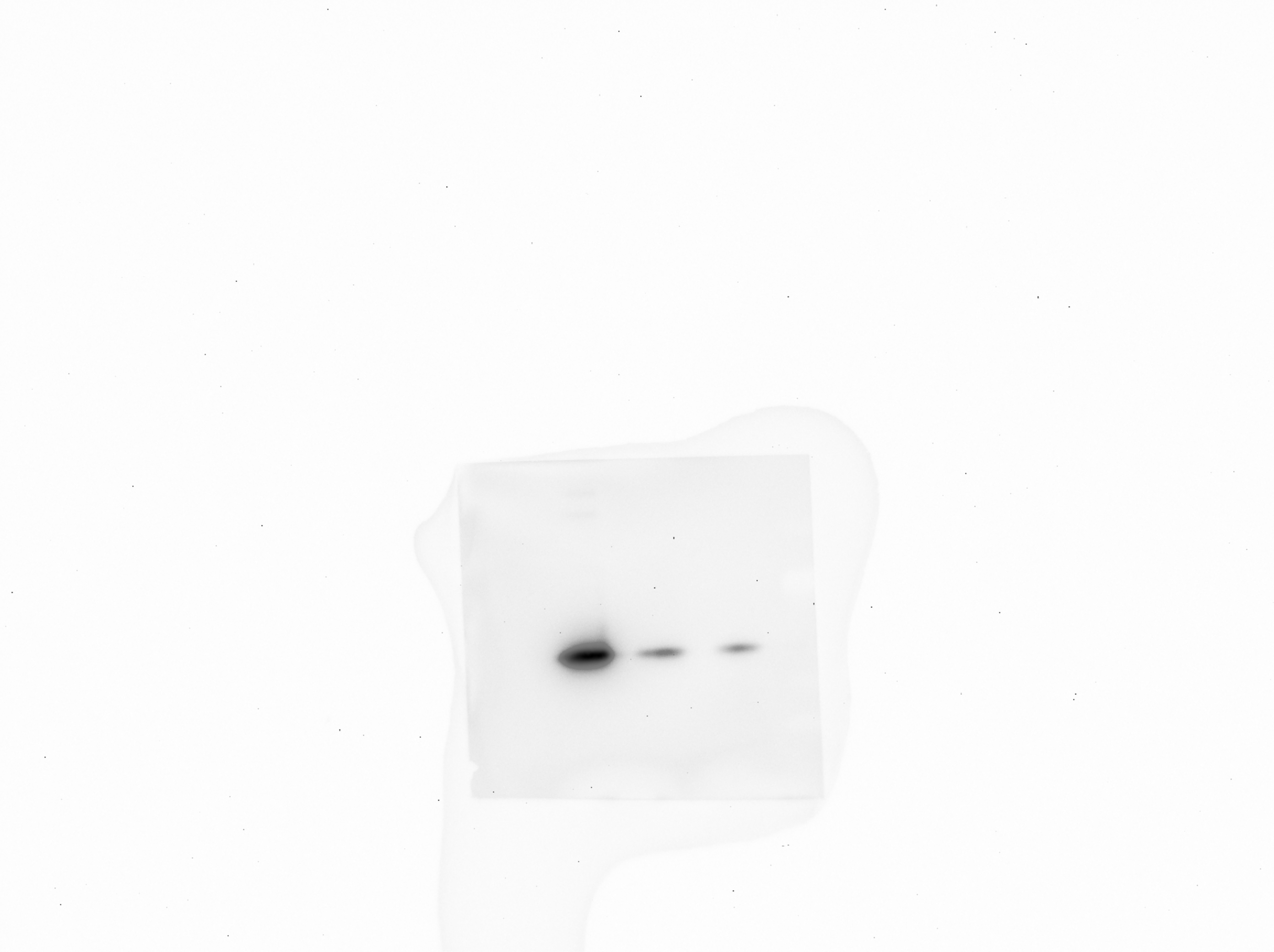

Supplement: Figure 7—source data 3. [file elife-84139-fig7-data3.zip › Figure 7 - source data 3. Western-blot uncropped membranes/Full blot Cav3.tif]

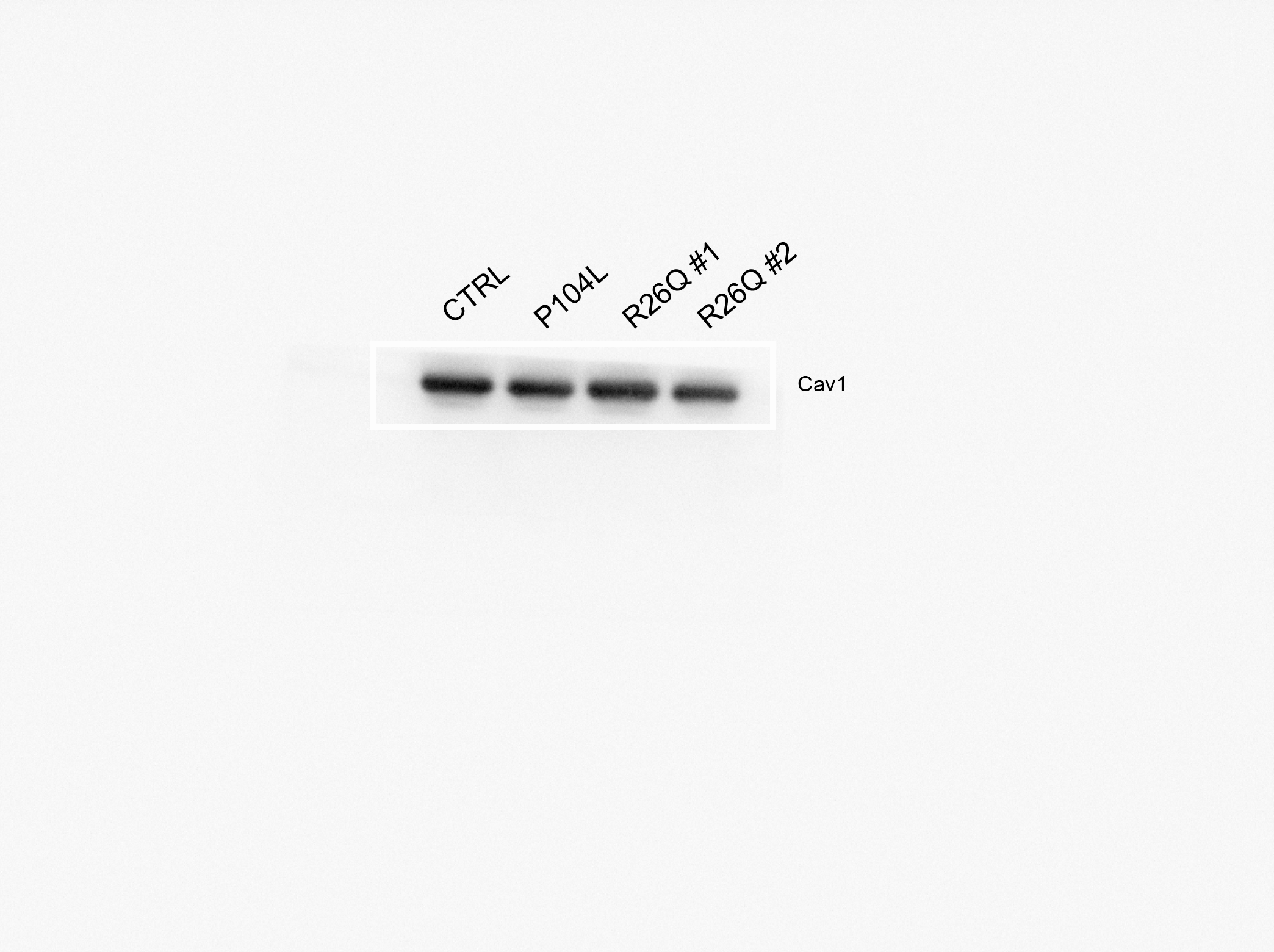

Supplement: Figure 8—figure supplement 1—source data 2. [file elife-84139-fig8-figsupp1-data2.zip › Figure 8 - figure supplement 1-source data 2. Western-blot uncropped membranes/Cav1.tif]

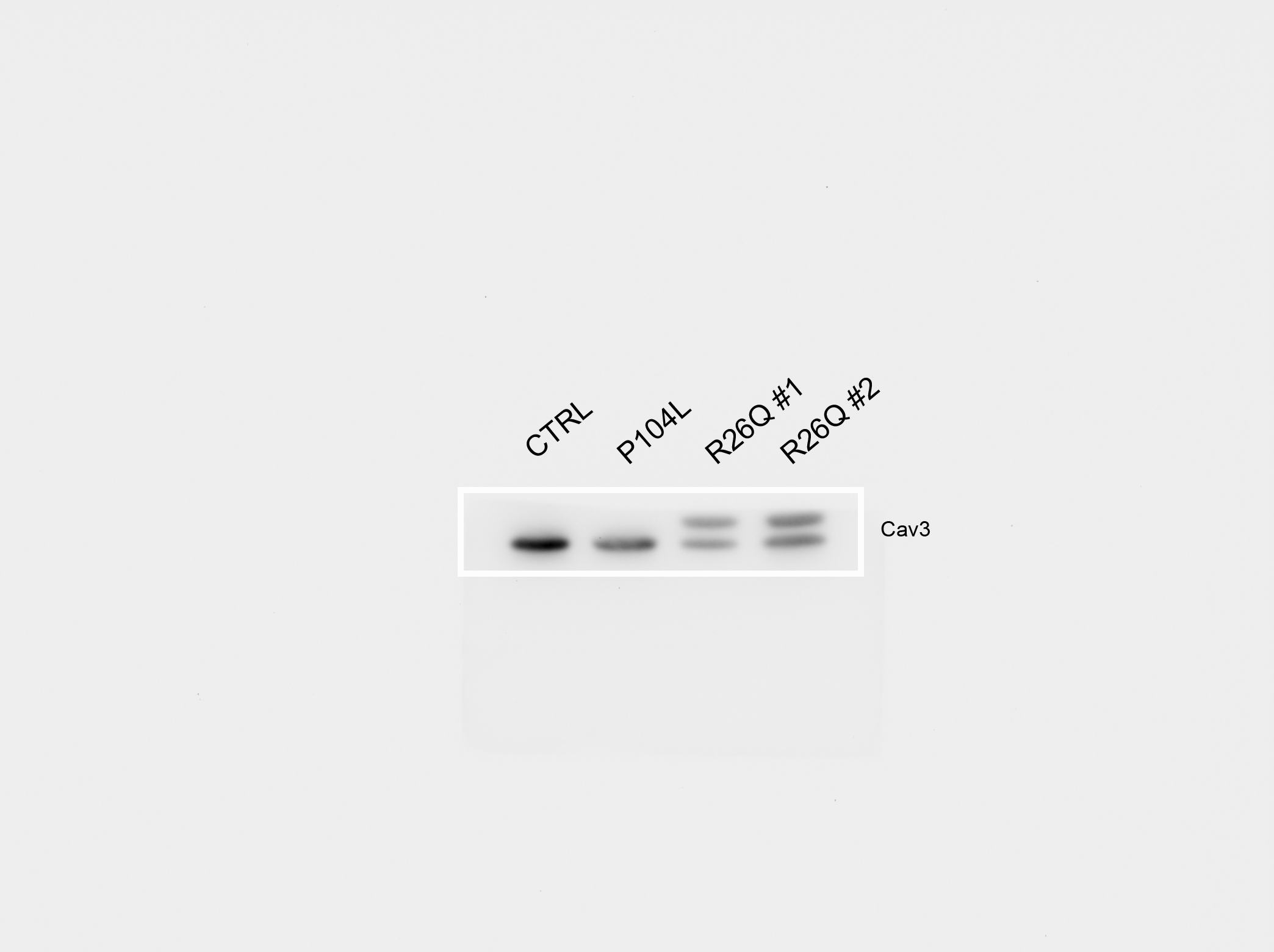

Supplement: Figure 8—figure supplement 1—source data 2. [file elife-84139-fig8-figsupp1-data2.zip › Figure 8 - figure supplement 1-source data 2. Western-blot uncropped membranes/Cav3.tif]

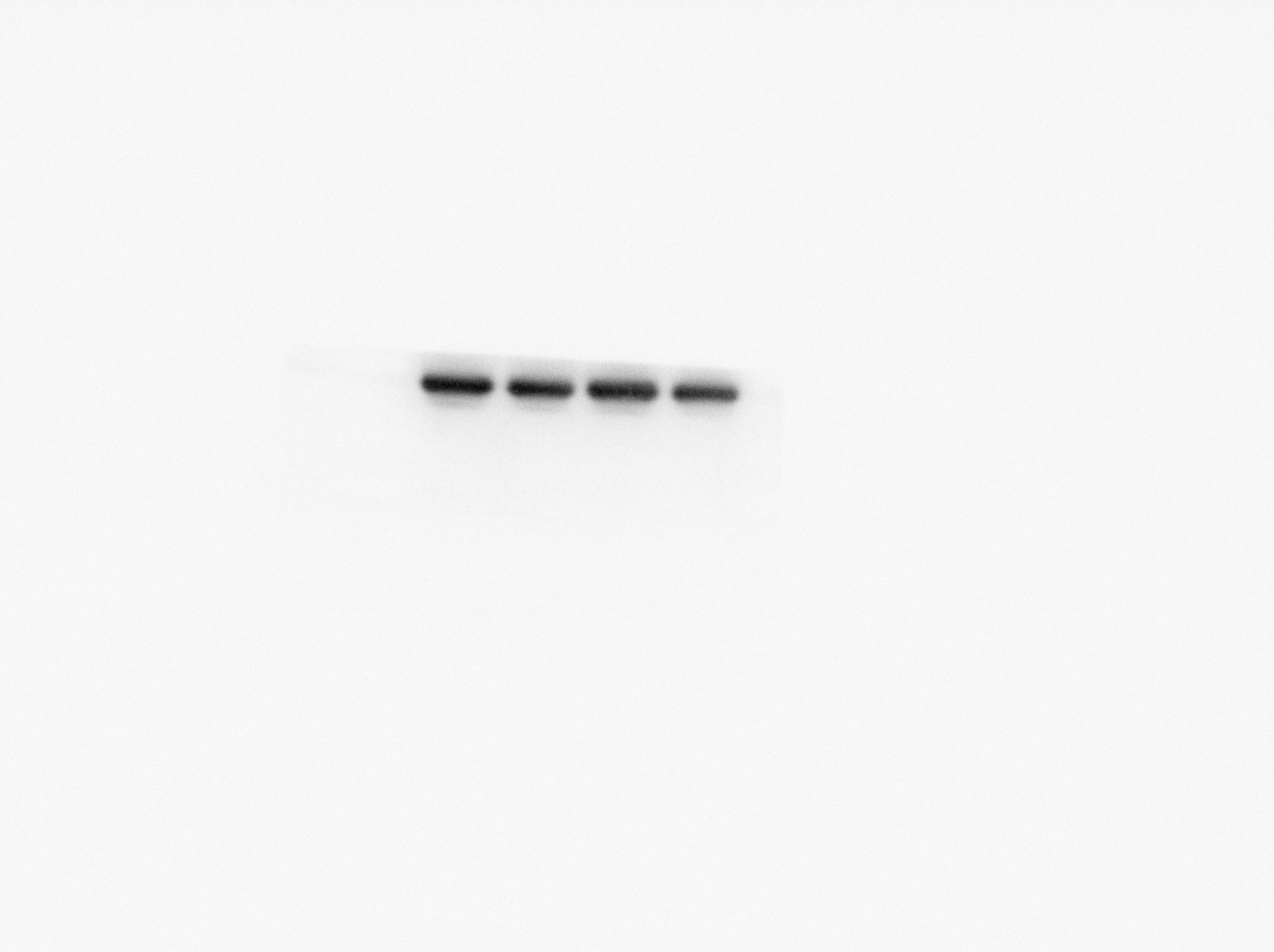

Supplement: Figure 8—figure supplement 1—source data 2. [file elife-84139-fig8-figsupp1-data2.zip › Figure 8 - figure supplement 1-source data 2. Western-blot uncropped membranes/full blot Cav1.tif]

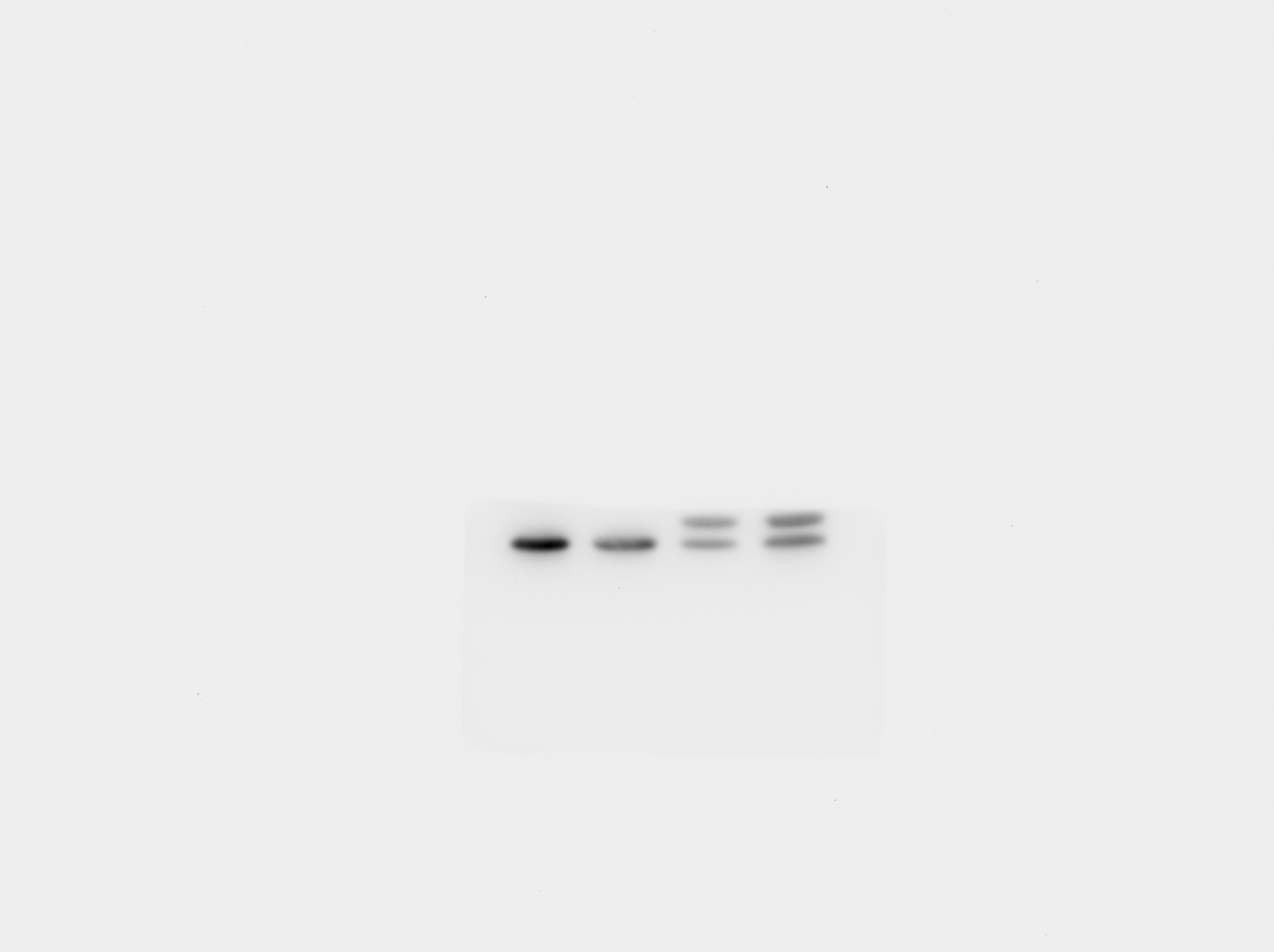

Supplement: Figure 8—figure supplement 1—source data 2. [file elife-84139-fig8-figsupp1-data2.zip › Figure 8 - figure supplement 1-source data 2. Western-blot uncropped membranes/Full blot Cav3.tif]

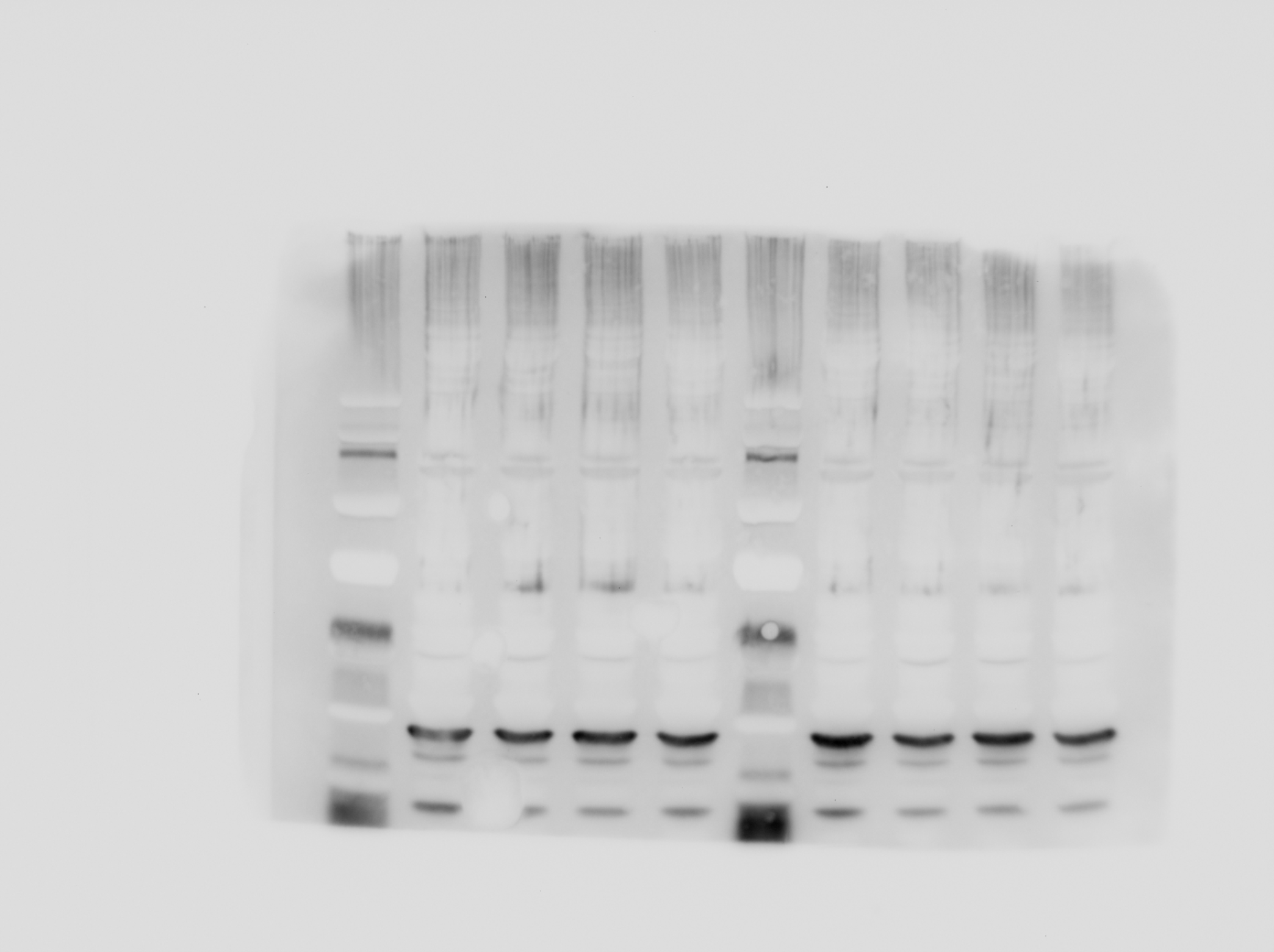

Supplement: Figure 8—figure supplement 1—source data 2. [file elife-84139-fig8-figsupp1-data2.zip › Figure 8 - figure supplement 1-source data 2. Western-blot uncropped membranes/Full blot GAPDH.tif]

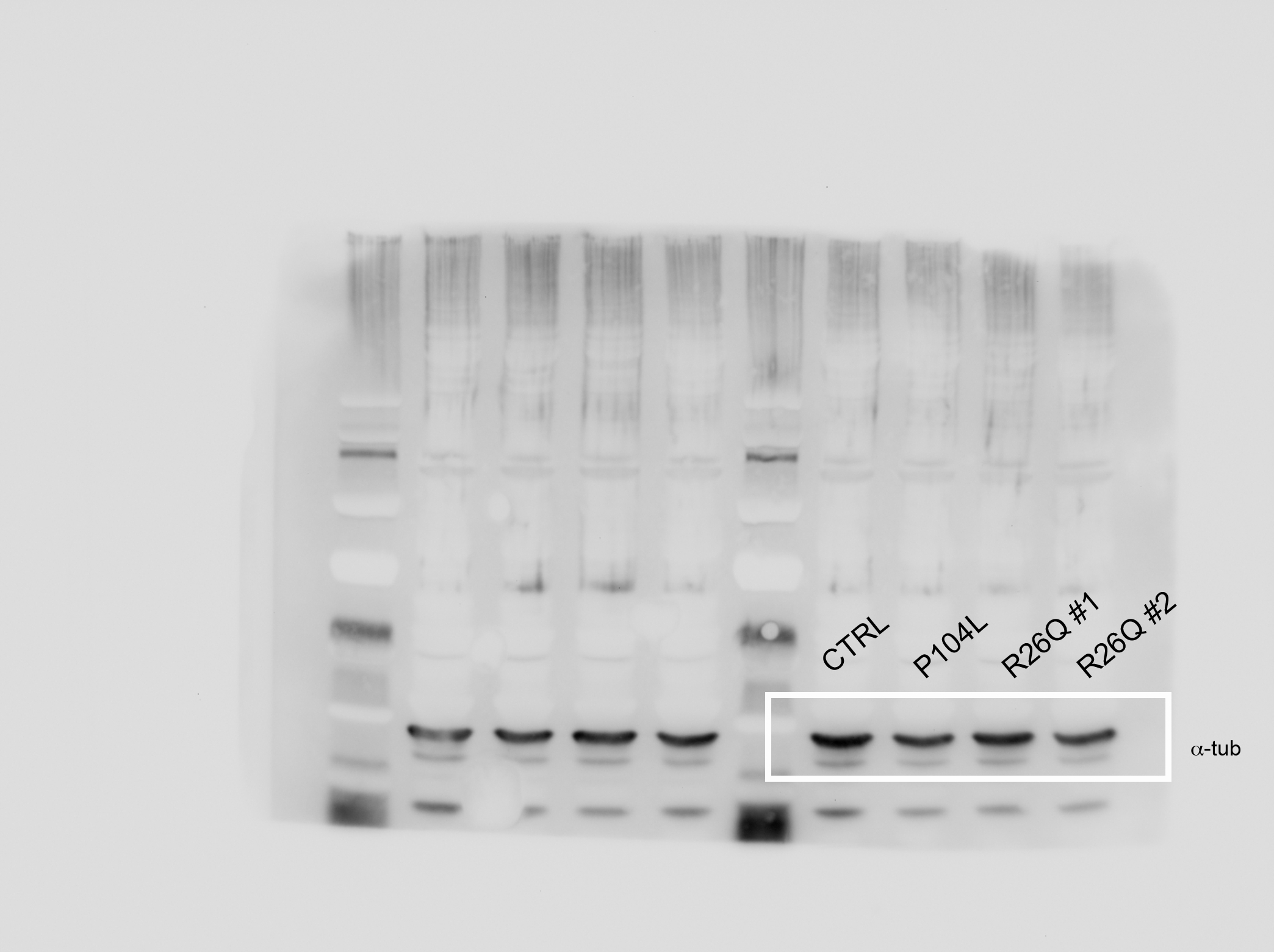

Supplement: Figure 8—figure supplement 1—source data 2. [file elife-84139-fig8-figsupp1-data2.zip › Figure 8 - figure supplement 1-source data 2. Western-blot uncropped membranes/GAPDH.tif]

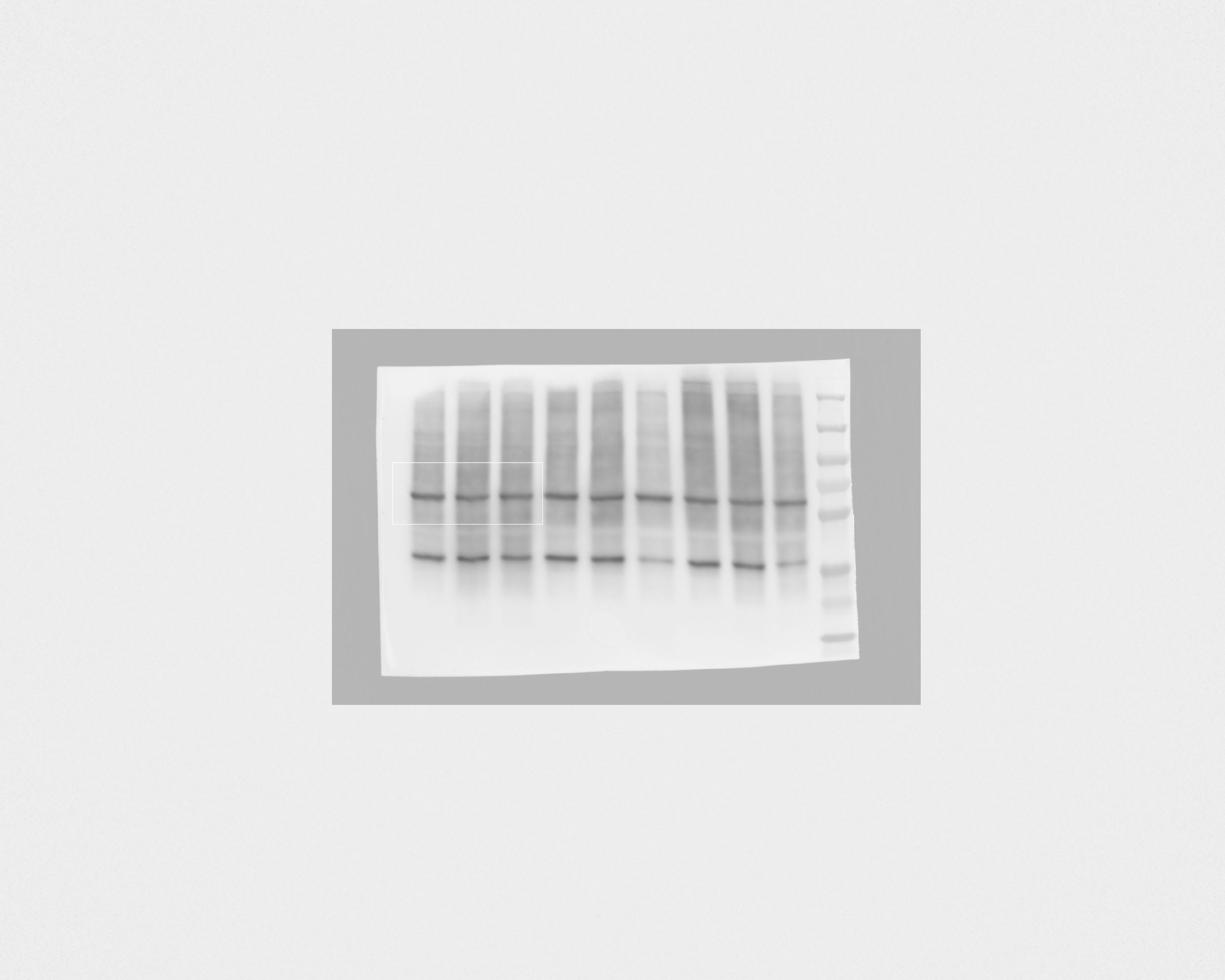

Supplement: Figure 8—figure supplement 1—source data 3. [file elife-84139-fig8-figsupp1-data3.zip › Figure 8 - figure supplement 1-source data 3. Western-blot uncropped membranes/230315-stainfree-JPH2_05(Chemiluminescence)flat.tif]

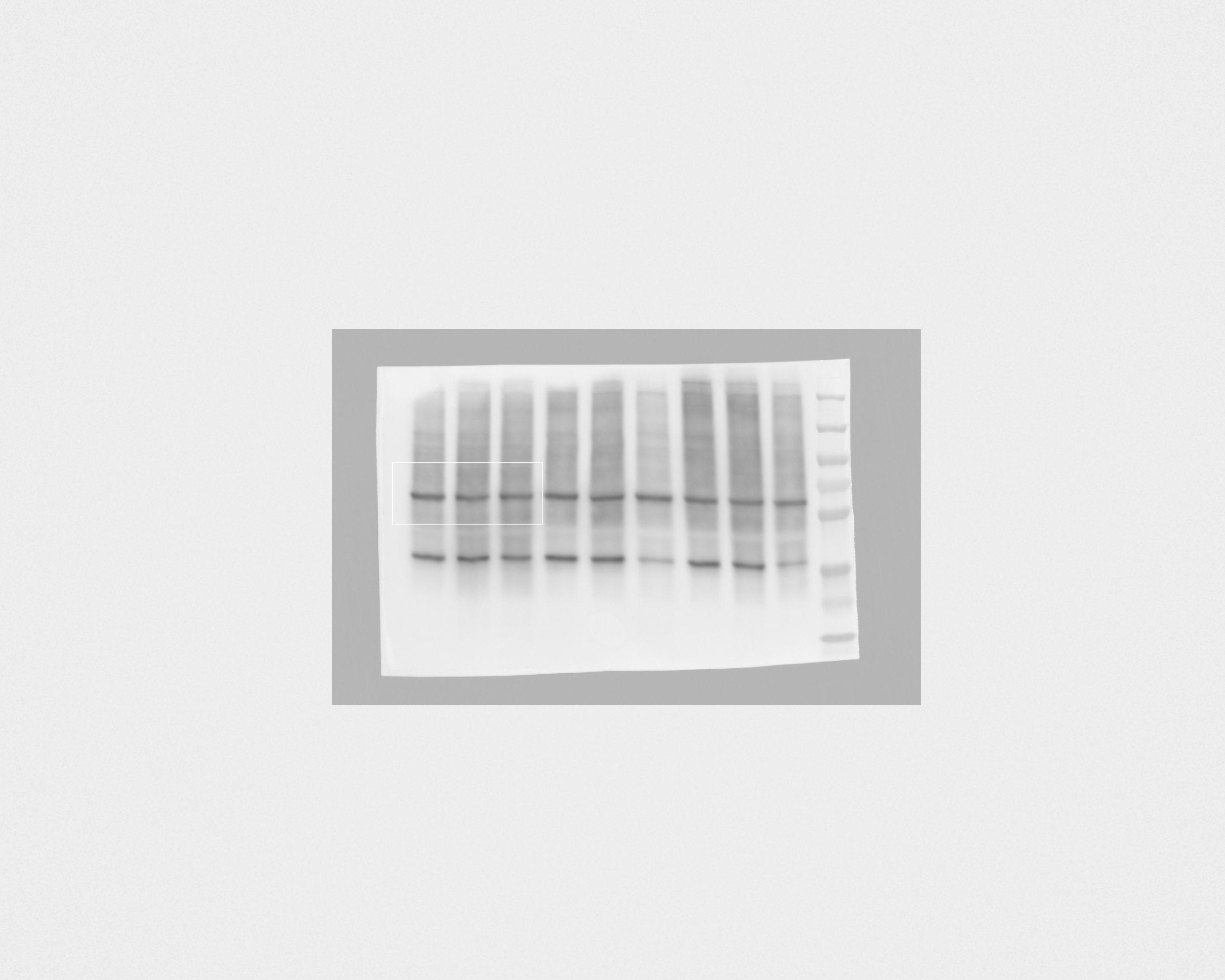

Supplement: Figure 8—figure supplement 1—source data 3. [file elife-84139-fig8-figsupp1-data3.zip › Figure 8 - figure supplement 1-source data 3. Western-blot uncropped membranes/230315-stainfree-JPH2_05(Chemiluminescence).tif]

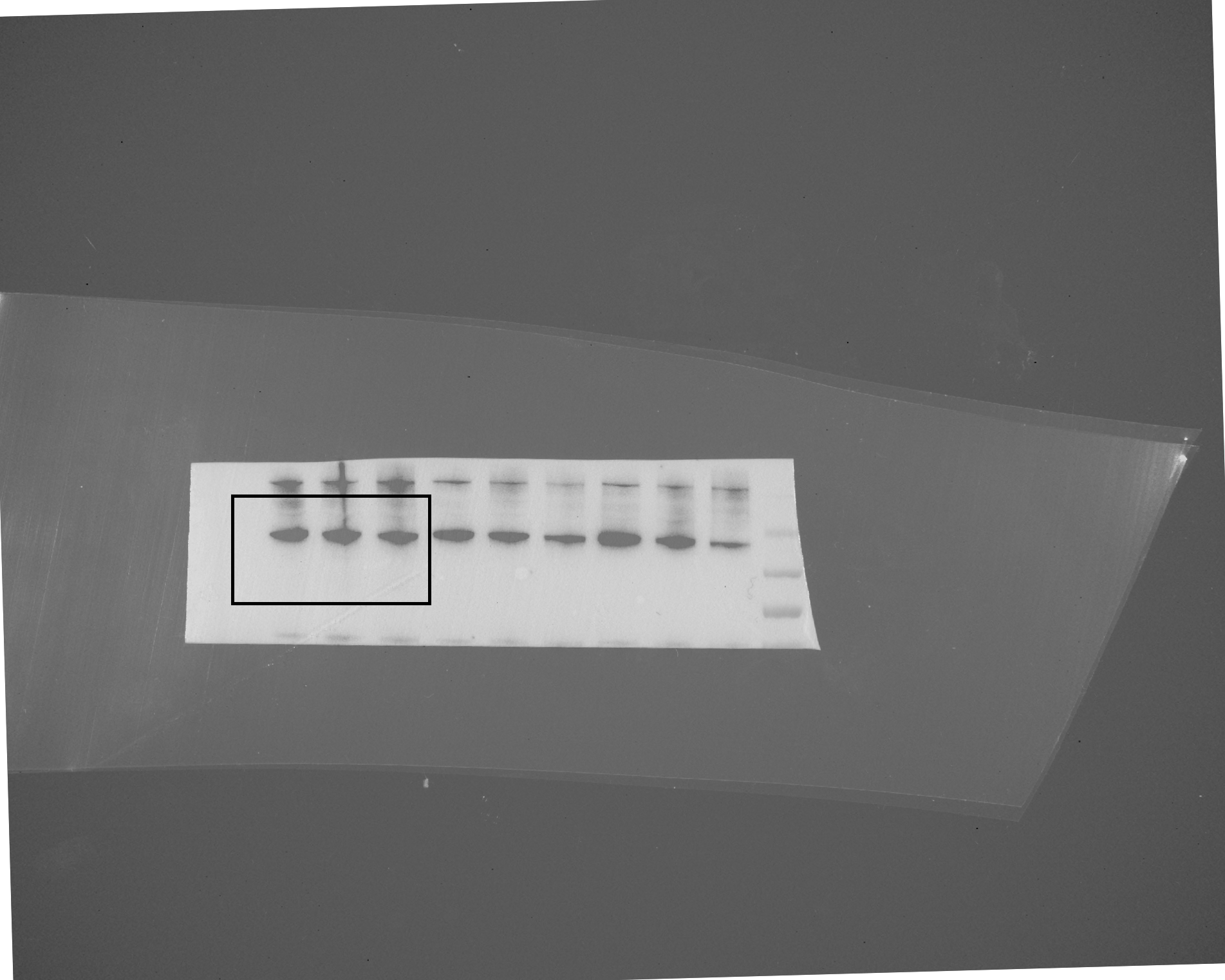

Supplement: Figure 8—figure supplement 1—source data 3. [file elife-84139-fig8-figsupp1-data3.zip › Figure 8 - figure supplement 1-source data 3. Western-blot uncropped membranes/230217-dhpr tub_2(Chemiluminescence).tif]

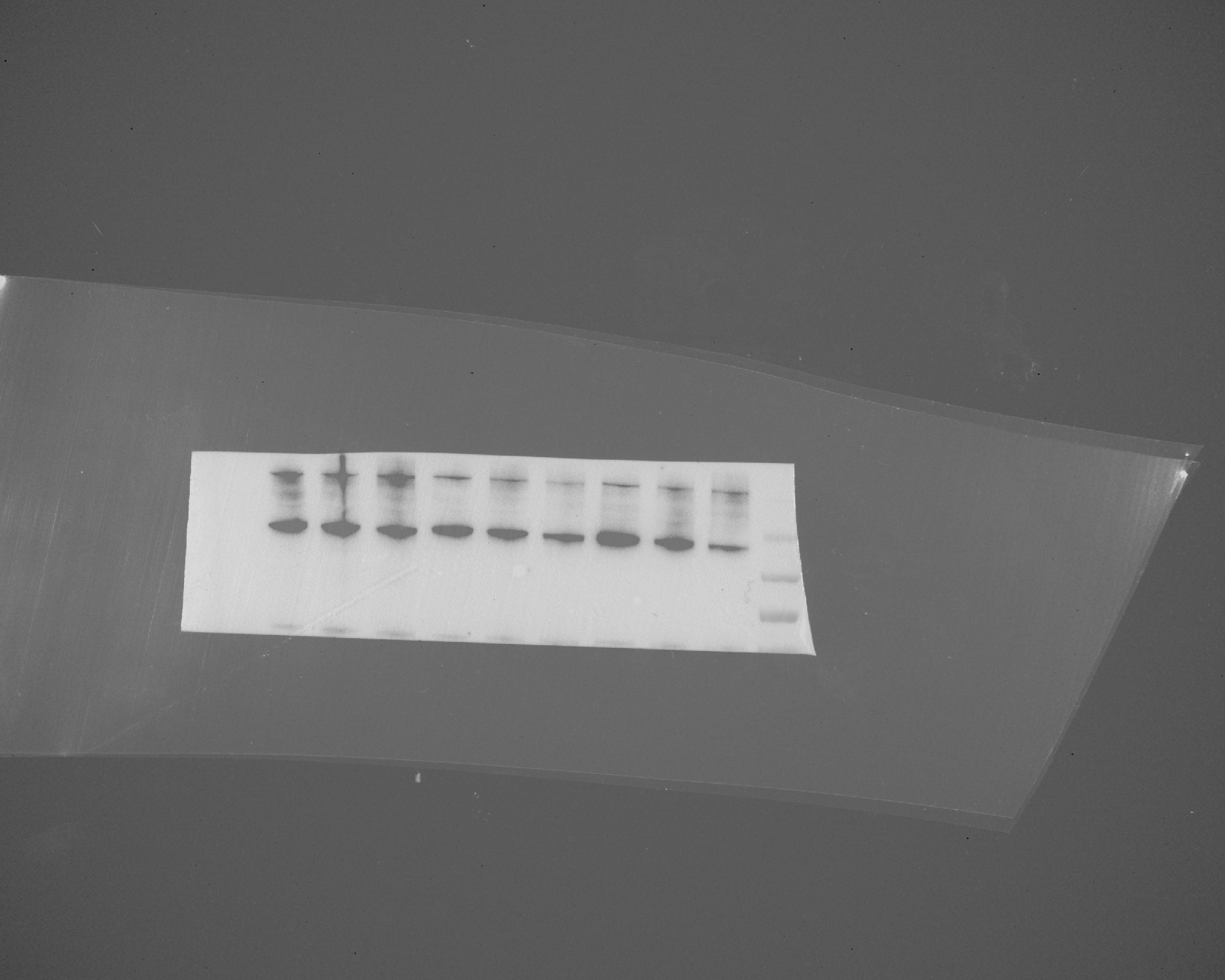

Supplement: Figure 8—figure supplement 1—source data 3. [file elife-84139-fig8-figsupp1-data3.zip › Figure 8 - figure supplement 1-source data 3. Western-blot uncropped membranes/230217-dhpr tub_4(Composite).tif]

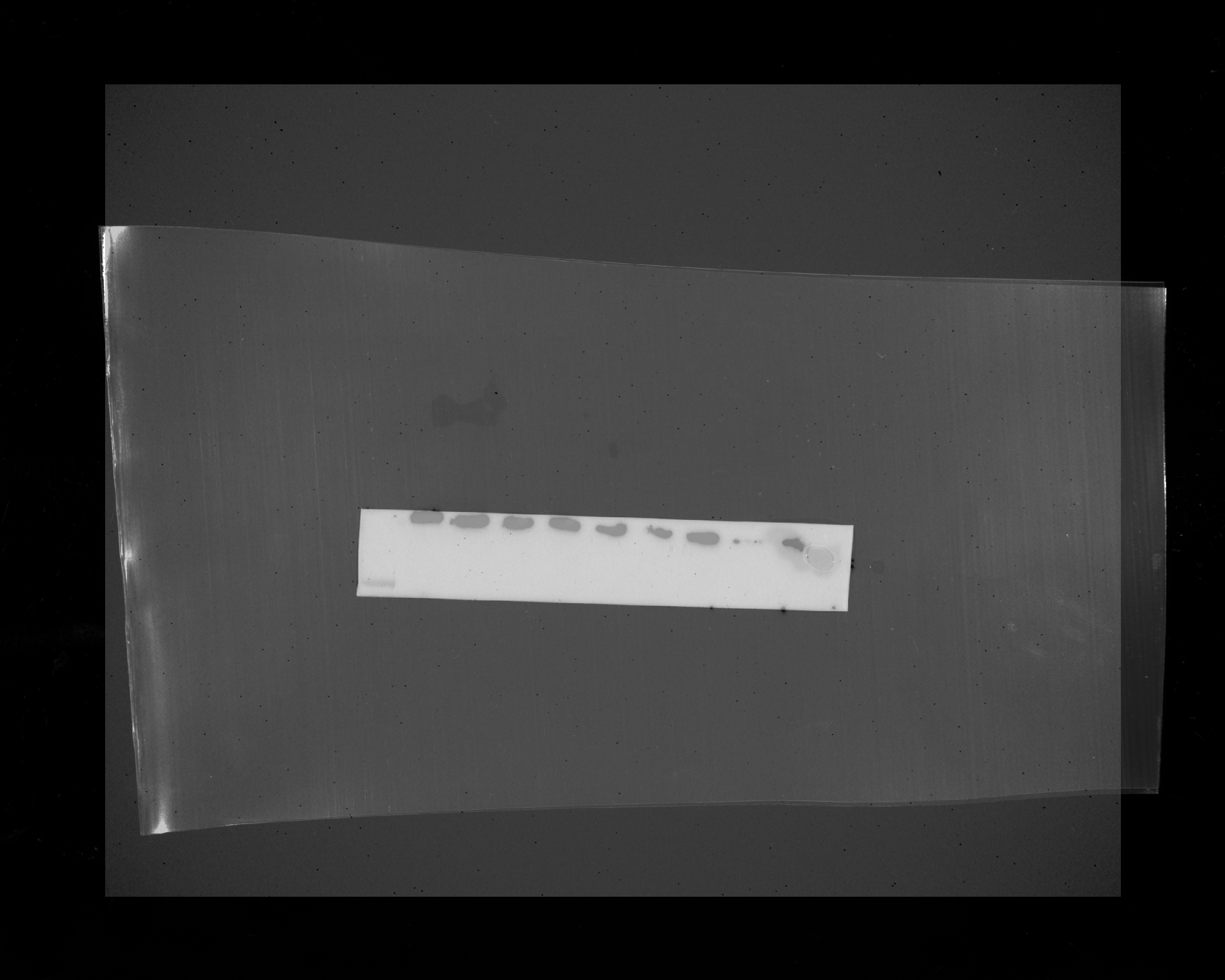

Supplement: Figure 8—figure supplement 1—source data 3. [file elife-84139-fig8-figsupp1-data3.zip › Figure 8 - figure supplement 1-source data 3. Western-blot uncropped membranes/230217-ryr tub_2(Colorimetric).tif]

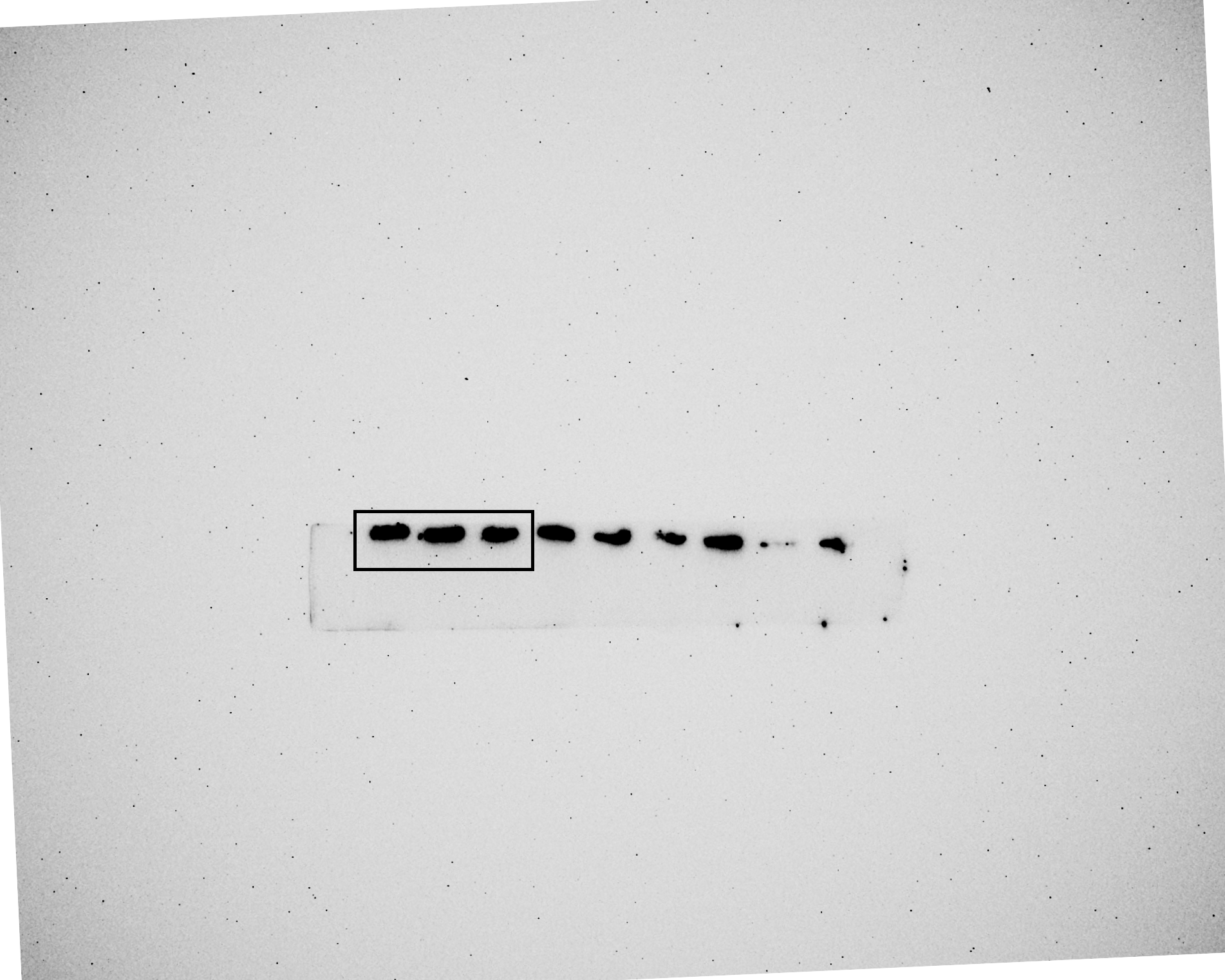

Supplement: Figure 8—figure supplement 1—source data 3. [file elife-84139-fig8-figsupp1-data3.zip › Figure 8 - figure supplement 1-source data 3. Western-blot uncropped membranes/230217-ryr tub_3(Chemiluminescence).tif]

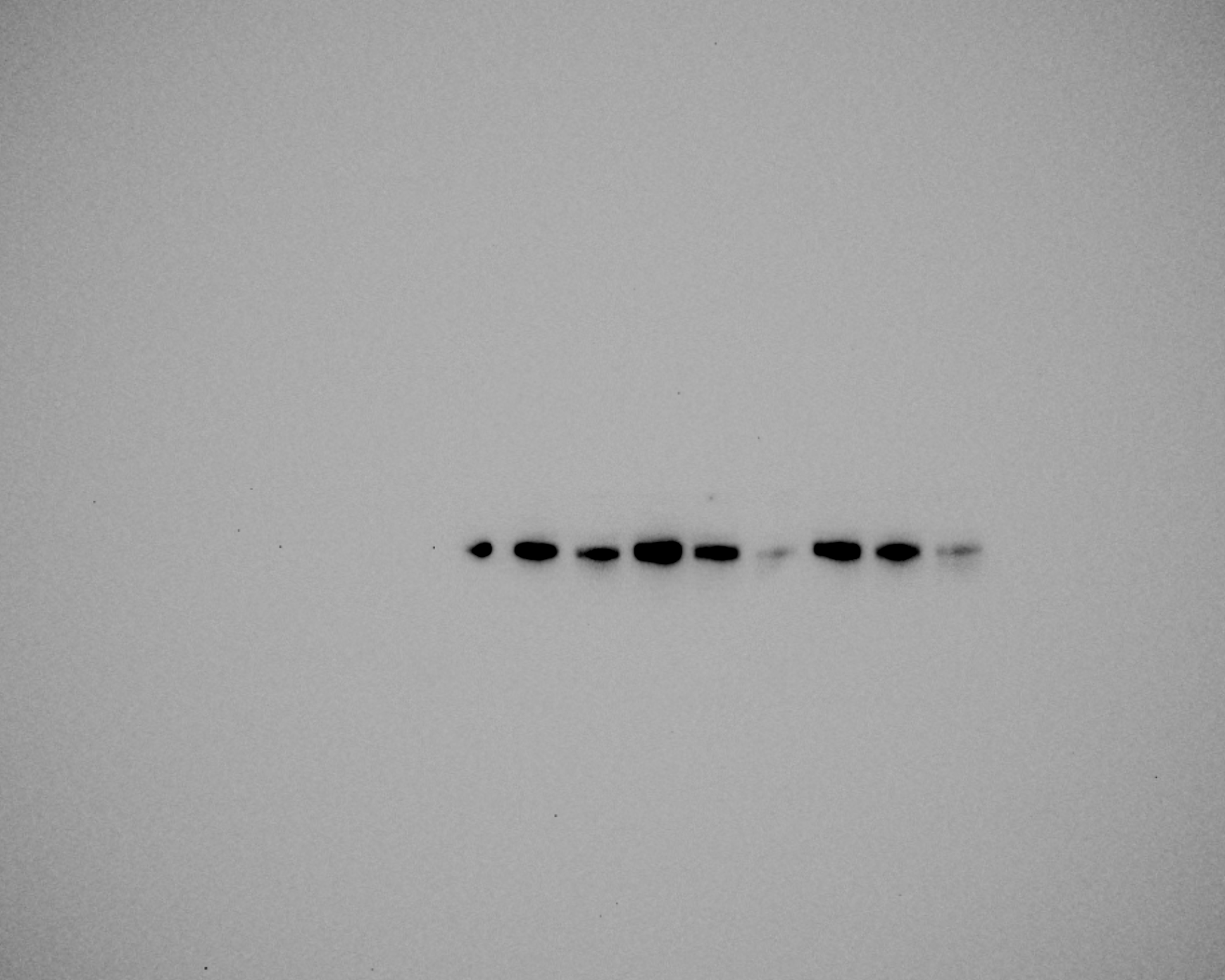

Supplement: Figure 8—figure supplement 1—source data 3. [file elife-84139-fig8-figsupp1-data3.zip › Figure 8 - figure supplement 1-source data 3. Western-blot uncropped membranes/230217-tubD(Chemiluminescence).tif]

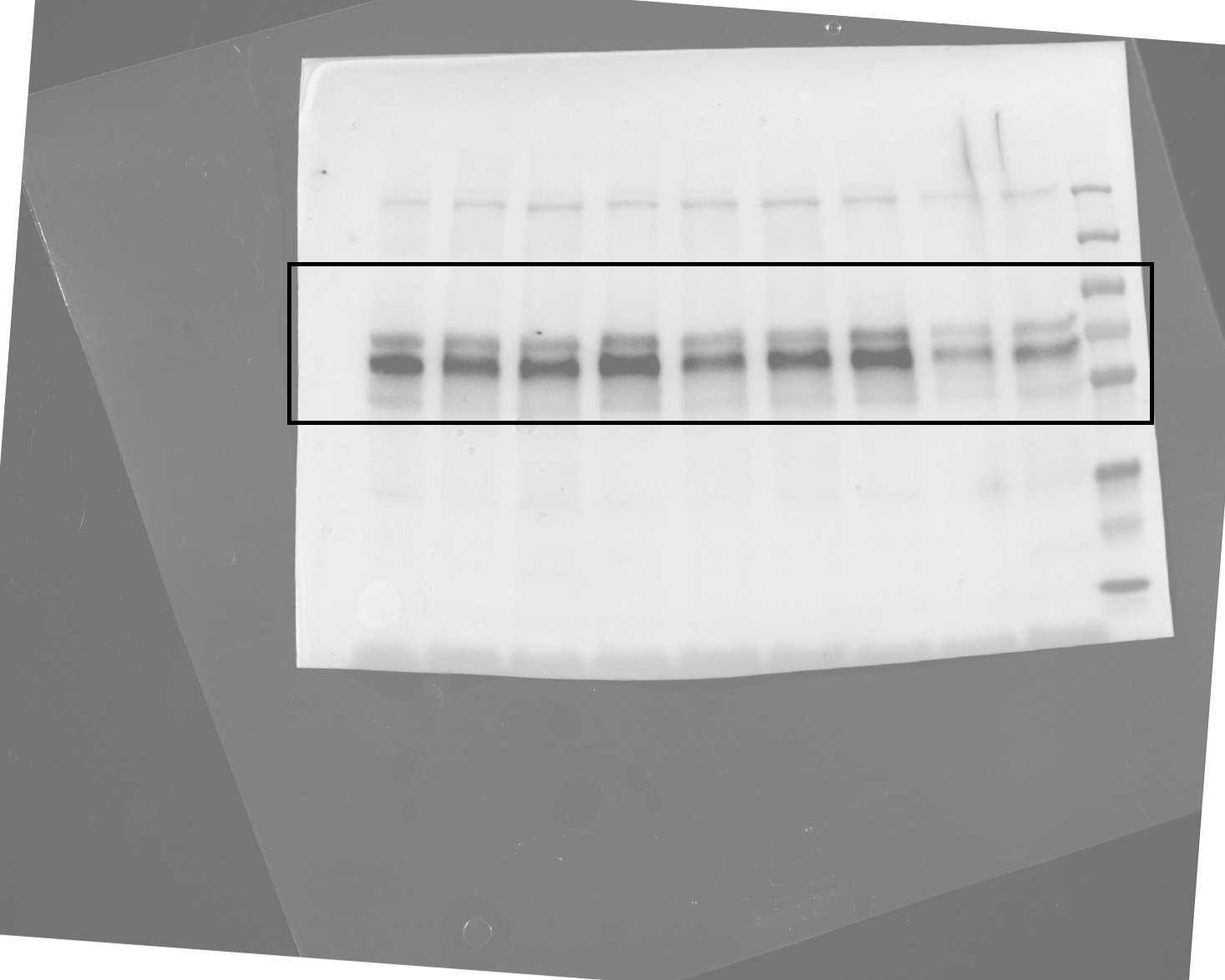

Supplement: Figure 8—figure supplement 1—source data 3. [file elife-84139-fig8-figsupp1-data3.zip › Figure 8 - figure supplement 1-source data 3. Western-blot uncropped membranes/230223-stainfree-bin1_01(Chemiluminescence).tif]

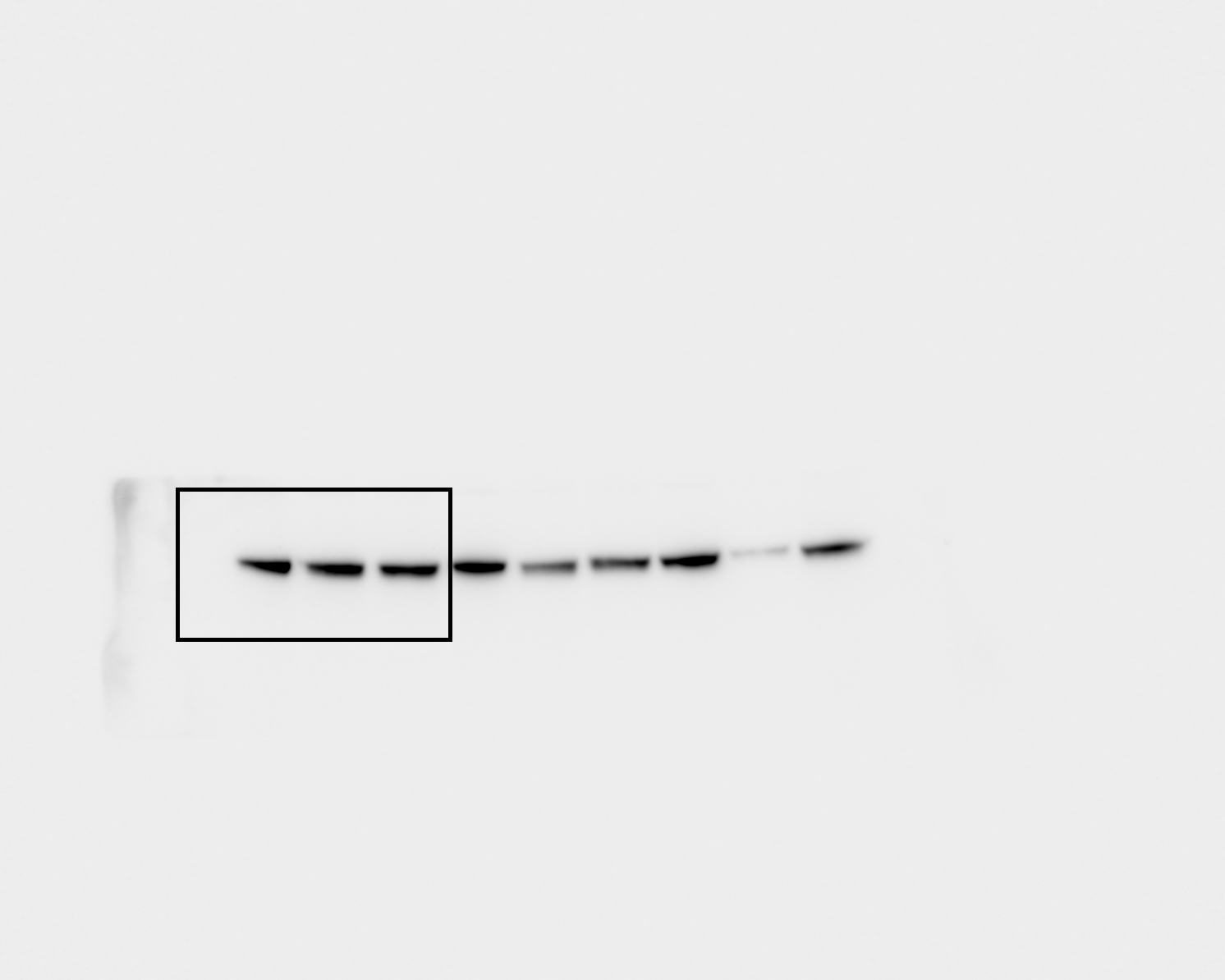

Supplement: Figure 8—figure supplement 1—source data 3. [file elife-84139-fig8-figsupp1-data3.zip › Figure 8 - figure supplement 1-source data 3. Western-blot uncropped membranes/230223-tubulin_03(Chemiluminescence).tif]
